# ﻿Phylogeography and genetic diversity of the Japanese mud shrimp *Upogebiamajor* (Crustacea, Decapoda, Upogebiidae): Natural or anthropogenic dispersal?

**DOI:** 10.3897/zookeys.1182.105030

**Published:** 2023-10-19

**Authors:** Kyosuke Kitabatake, Kentaro Izumi, Natsuko I. Kondo, Kenji Okoshi

**Affiliations:** 1 Graduate School of Science, Toho University, 2-2-1 Miyama, Funabashi, Chiba 274-8510, Japan Toho University Funabashi Japan; 2 Faculty and Graduate School of Education, Chiba University, 1-33 Yayoi-cho, Inage-ku, Chiba-shi, Chiba 263-8522, Japan Chiba University Chiba-shi Japan; 3 Biodiversity Division, National Institute for Environmental Studies, 16-2 Onogawa, Tsukuba, Ibaraki 305-8506, Japan National Institute for Environmental Studies Tsukuba Japan

**Keywords:** Artificial introduction, Geographical distribution, Molecular phylogenetics, Morphological analysis, Ocean current, Topographic change, *Upogebiamajor*

## Abstract

*Upogebiamajor* (De Haan, 1841) is known for forming huge burrows in sandy, intertidal areas that can extend to depths of over 2 m. Despite its widespread distribution in East Asia and Russia, the genetic relatedness of its regional populations remains uncertain, likely owing to difficulties in specimen collection. Therefore, to appraise the phylogeographic patterns, genetic diversity, and morphological variety of *U.major*, the mitochondrial DNA of specimens collected from Japan, Korea and China were subjected to molecular phylogenetic analyses of COI genes, alongside morphological assessment. As a result, we discovered four principal groups; of these, Group 1 consisted predominantly of Japanese specimens, while Groups 3 and 4 were interpreted as having originated from the continent. Group 2 exhibited genetic segregation from both continental and Japanese descent. Group 1 mostly comprising Japanese specimens implies that the planktonic larvae of *U.major* were disseminated north and south by ocean currents encompassing the Japanese archipelago. In contrast, individuals probably originating from the continent were discovered in Lake Notoro, Hokkaido and Matsukawa-ura, Fukushima in northeastern Japan, indicating possible introduction from the continent through ocean currents or unintentional introduction with other organisms imported. Additionally, one of the specimens collected from Matsukawa-ura exhibited significant genetic and morphological differences from other specimens, suggesting the possibility of being a subspecies. The outcomes of this study not only offer valuable insights into the origins of distribution of *U.major* but also introduce a novel challenge of assessing the coexistence of two routes: natural and anthropogenic dispersion.

## ﻿Introduction

The *Upogebia* genus constitutes a cluster of crustaceans, commonly referred to as mud shrimp, which inhabit all corners of the world and encompass approximately 280 species. Amongst these, the Japanese archipelago harbours 13 species (Komai 2020). Several of these species are characterised by their capacity to construct extensive burrows exceeding depths of 1 m. In particular, *Upogebiamajor* (De Haan 1841), is renowned for its ability to create large Y-shaped burrow, some exceeding depths of 2 m, in substrates such as slightly silty sand within the shallow waters of the coastal regions of the Japanese archipelago, Korean Peninsula, Shandong Peninsula, and the Russian coast facing the Sea of Japan (Kinoshita 2002; Itani 2004; Sakai 2006; Selin 2017; Kinoshita 2022). Collecting this species poses a considerable challenge because of its deep burrow habitat and poorly understood ecological characteristics. However, *U.major* is an active edificator that plays a pivotal role in shaping the qualitative composition and abundance of benthic organisms (Kinoshita et al. 2008; Hong 2013; Kinoshita 2022). The abundance of *U.major* and its bioturbation activities have made it the subject of increasing scientific interest as an essential contributor to the structure of benthic communities. In particular, this species has garnered attention as a host for numerous symbiotic organisms within its burrows and on its body (Kinoshita et al. 2008; Seike and Goto 2020; Shiozaki and Itani 2020). Similar research has been conducted on closely-related species such as *Upogebiayokoyai* (Henmi and Itani 2014, 2021). Larger specimens of *U.major* are commonly harvested and consumed in Western Japan and South Korea (Sato 2000; Ngoc-Ho 2001; Hong 2013; Das et al. 2017), whereas smaller specimens are employed as fishing bait in Japan (Kitabatake, personal observation). Despite extensive research on the ecology, environment, symbiotic relationships and economic implications of *U.major*, phylogenetic and genetic investigations are limited and the origins of local populations remain largely unknown despite their wide distribution in East Asia and Russia. Recently, genetic analyses utilising complete mitochondrial genomes have elucidated the evolutionary history of *U.major* at the familial and generic levels, indicating its close affiliation with members of the Thalassinidae family, with *U.yokoyai* as its closest relative (Sun and He 2021). Nonetheless, intraspecific research on *U.major* remains limited and a comprehensive assessment of its phylogeography and genetic diversity has yet to be accomplished. These data could shed light on the migration and dispersal processes that underlie the geographical distribution of this species.

The study of the phylogeography and genetic diversity of benthic organisms inhabiting coastal marine waters has been extensively researched on a global scale. For instance, *Oratosquillaoratoria* and *Eriocheirjaponica*, crustaceans that inhabit the coast of China facing the East China Sea, display marked genetic divergence between the northern and southern regions of China, primarily because of topographic changes associated with the opening of the Sea of Japan (Zhang et al. 2014). The echinoderm *Ophiurasarsii*, which is widely distributed throughout the Arctic and sub-Arctic regions of the Atlantic and Pacific Oceans, exhibits a marked genetic diversity in the Barents Sea. Their population and spatial expansion are hypothesised to have taken place in the Barents Sea during the Bølling–Allerød interglacial epoch of the melting glacial period in the western margin region of the Barents Sea (Genelt-Yanovskiy et al. 2021). Furthermore, the unintentional migration of the Caribbean polychaete *Branchiommabairdi*, facilitated by commercial shipping, led to its discovery in Tunisia, where the genetic features of native individuals were similar to those of introduced individuals (Khedhri et al. 2017). Several analogous studies have been documented around the Japanese archipelago, albeit their quantity is limited. For example, *Turbosazae*, a rocky reef inhabitant and *Batillariacumingii*, a mud flat dweller, exhibit distinct haplotypes on the Sea of Japan and Pacific sides of the Japanese archipelago (Kojima et al. 1997, 2000, 2004; Yanagimoto et al. 2022). This differentiation is attributed to gene flow via separate ocean currents on the Sea of Japan and the Pacific. Additionally, the crustacean *Tachypleustridentatus*, distributed from south-eastern to western Japan, was found in Mikawa Bay in central Japan with a haplotype of Chinese origin, likely originating from individuals introduced from China for commercial purposes and is now considered an alien species (Nishida et al. 2015). Thus, a multitude of prior investigations regarding phylogeography and genetic diversity have demonstrated that the geographical distribution of marine benthos results from the complex interplay of physical and anthropogenic factors. Conversely, research on epifaunal organisms, which are readily collected, has taken centre stage, whereas enquiries into infaunal species, such as *U.major*, which are deeply embedded in sediments, appear to be scarce.

In this study, molecular phylogenetic analyses were performed on *U.major* specimens collected from Japan, Korea and China. A phylogenetic tree and haplotype network, based on the cytochrome *c* oxidase subunit I (COI) of mitochondrial DNA, were constructed to reveal detailed interspecific relationships. Furthermore, the calculation of pairwise population differentiation (*F_ST_*) and analysis of molecular variance (AMOVA) were performed to scrutinise the genetic variation amongst populations. Morphological measurements were conducted to examine the possible correlations between genetic variation and morphological characteristics. Through these examinations, the phylogeography and genetic diversity of *U.major* were evaluated and the physical and anthropogenic factors responsible for the genetic differentiation of the species in the vicinity of the Japanese archipelago were discussed. The findings of this study provide crucial insights into the geographical origins of *U.major*.

## ﻿Materials and methods

### ﻿Sampling sites

**Figure 1.**
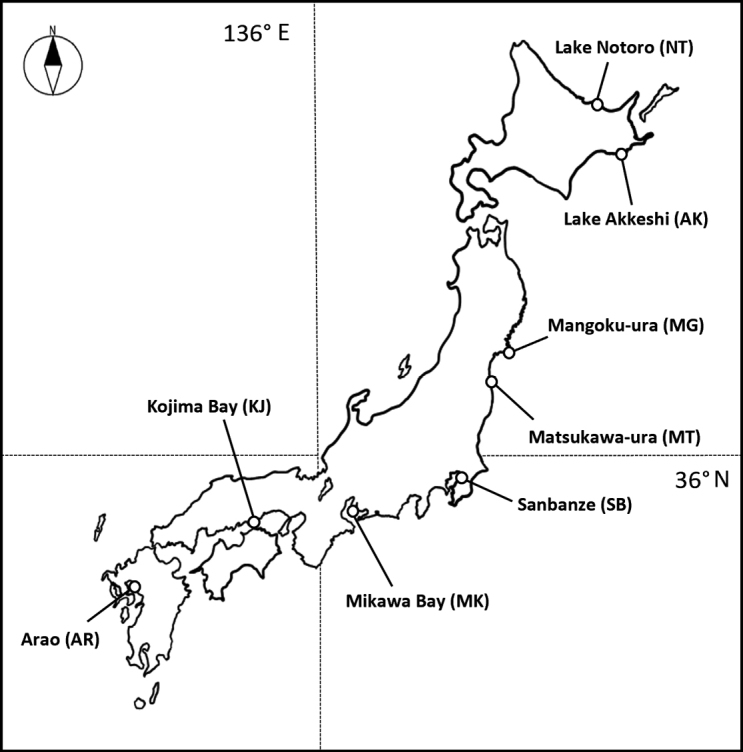
Map of the sampling sites for *Upogebiamajor* examined in this study.

Samples of *U.major* were collected during the period of September 2021 to July 2022 from five sites in east and northern Japan (Fig. 1) by brush and yabbie pump (Poseidon, Aichi, Japan) during low tide. The sampling sites are Lake Notoro (NT) and Lake Akkeshi, Hokkaido (AK), Mangoku-ura, Lagoon (or Inlet), Miyagi (MG), Matsukawa-ura, Lagoon, Fukushima (MT) and Sanbanze, Chiba (SB). *U.major* lived at high densities (at least 20 to over 100 burrows per square metre) at the AK sites. In addition, *U.major* purchased from the following areas at Mikawa Bay, Aichi (MK), Kojima Bay, Okayama (KJ) and Arao, Kumamoto (AR) were stored at -25 °C immediately after collection. A total of 53 samples were analysed: nine from NT, three from AK, seven from MG, seven from MT, five from SB, seven from MK, 11 from KJ and four from AR, respectively (Table 1).

**Table 1.** (A) Provides a comprehensive representation of *U.major*, a Japanese species, that was obtained for this study. The depiction encompasses a multitude of details, including sample code, collection site, date of collection, isolation source and gene. (B) Depicts nucleotide sequence data obtained from GenBank, including crucial information such as sample code, species, accession number and elaborate information concerning collection location, literature and gene. The collected location of SK-03-05 was procured and has been verified by GBIF (Global Biodiversity Information Facility) (https://www.gbif.org/ja/species/2222995) on the grounds of the voucher number itemised in the registration information of the Accession Number.

A	Sample code	Collected location	Geographical coordinates	Collected date	Isolation source	Gene	Accession No.
	NT-01	Lake Notoro, Hokkaido	44.0425°N, 144.1704°E	29.july.22	Abdomen tissue	COI	LC761102
	NT-02	Lake Notoro, Hokkaido	44.0425°N, 144.1704°E	29.july.22	Abdomen tissue	COI	LC761103
	NT-03	Lake Notoro, Hokkaido	44.0425°N, 144.1704°E	29.july.22	Abdomen tissue	COI	LC761104
	NT-04	Lake Notoro, Hokkaido	44.0425°N, 144.1704°E	29.july.22	Abdomen tissue	COI	LC761105
	NT-05	Lake Notoro, Hokkaido	44.0425°N, 144.1704°E	29.july.22	Abdomen tissue	COI	LC761106
	NT-06	Lake Notoro, Hokkaido	44.0425°N, 144.1704°E	29.july.22	Abdomen tissue	COI	LC761107
	NT-07	Lake Notoro, Hokkaido	44.0425°N, 144.1704°E	29.july.22	Abdomen tissue	COI	LC761108
	NT-08	Lake Notoro, Hokkaido	44.0425°N, 144.1704°E	29.july.22	Abdomen tissue	COI	LC761109
	NT-09	Lake Notoro, Hokkaido	44.0425°N, 144.1704°E	29.july.22	Abdomen tissue	COI	LC761110
	AK-01	Lake Akkeshi, Hokkaido	43.0256°N, 144.8792°E	28.july.22	Abdomen tissue	COI	LC761111
	AK-02	Lake Akkeshi, Hokkaido	43.0256°N, 144.8792°E	28.july.22	Abdomen tissue	COI	LC761112
	AK-03	Lake Akkeshi, Hokkaido	43.0256°N, 144.8792°E	28.july.22	Abdomen tissue	COI	LC761113
	MG-01	Mangoku-ura, Miyagi	38.4185°N, 141.3820°E	22.March.22	Abdomen tissue	COI	LC761114
	MG-02	Mangoku-ura, Miyagi	38.4185°N, 141.3820°E	22.March.22	Abdomen tissue	COI	LC761115
	MG-03	Mangoku-ura, Miyagi	38.4185°N, 141.3820°E	22.March.22	Abdomen tissue	COI	LC761116
	MG-04	Mangoku-ura, Miyagi	38.4185°N, 141.3820°E	22.March.22	Abdomen tissue	COI	LC761117
	MG-05	Mangoku-ura, Miyagi	38.4185°N, 141.3820°E	19.May.22	Abdomen tissue	COI	LC761118
	MG-06	Mangoku-ura, Miyagi	38.4185°N, 141.3820°E	19.May.22	Abdomen tissue	COI	LC761119
	MG-07	Mangoku-ura, Miyagi	38.4185°N, 141.3820°E	19.May.22	Abdomen tissue	COI	LC761120
	MT-01	Matsukaura, Fukushima	37.8128°N, 140.9723°E	21.September.21	Manus tissue	COI	LC761121
	MT-02	Matsukaura, Fukushima	37.8128°N, 140.9723°E	21.September.21	Manus tissue	COI	LC761122
	MT-03	Matsukaura, Fukushima	37.8128°N, 140.9723°E	21.September.21	Manus tissue	COI	LC761123
	MT-04	Matsukaura, Fukushima	37.8128°N, 140.9723°E	20.May.22	Abdomen tissue	COI	LC761124
	MT-05	Matsukaura, Fukushima	37.8128°N, 140.9723°E	20.May.22	Abdomen tissue	COI	LC761125
	MT-06	Matsukaura, Fukushima	37.8128°N, 140.9723°E	20.May.22	Abdomen tissue	COI	LC761126
	MT-07	Matsukaura, Fukushima	37.8128°N, 140.9723°E	20.May.22	Abdomen tissue	COI	LC761127
	SB-01	Sanbanze, Chiba	35.6709°N, 139.9689°E	5.October.21	Manus tissue	COI	LC761128
	SB-02	Sanbanze, Chiba	35.6709°N, 139.9689°E	5.October.21	Manus tissue	COI	LC761129
	SB-03	Sanbanze, Chiba	35.6709°N, 139.9689°E	5.October.21	Manus tissue	COI	LC761130
	SB-04	Sanbanze, Chiba	35.6709°N, 139.9689°E	5.October.21	Manus tissue	COI	LC761131
	SB-05	Sanbanze, Chiba	35.6709°N, 139.9689°E	1.july.22	Abdomen tissue	COI	LC761132
	MK-01	Mikawa Bay, Aichi	Unknown	Purchased	Manus tissue	COI	LC761133
	MK-02	Mikawa Bay, Aichi	Unknown	Purchased	Manus tissue	COI	LC761134
	MK-03	Mikawa Bay, Aichi	Unknown	Purchased	Manus tissue	COI	LC761135
	MK-04	Mikawa Bay, Aichi	Unknown	Purchased	Manus tissue	COI	LC761136
	MK-05	Mikawa Bay, Aichi	Unknown	Purchased	Manus tissue	COI	LC761137
	MK-06	Mikawa Bay, Aichi	Unknown	Purchased	Abdomen tissue	COI	LC761138
	MK-07	Mikawa Bay, Aichi	Unknown	Purchased	Abdomen tissue	COI	LC761139
	KJ-01	Kojima Bay, Okayama	Unknown	Purchased	Manus tissue	COI	LC761140
	KJ-02	Kojima Bay, Okayama	Unknown	Purchased	Manus tissue	COI	LC761141
	KJ-03	Kojima Bay, Okayama	Unknown	Purchased	Manus tissue	COI	LC761142
	KJ-04	Kojima Bay, Okayama	Unknown	Purchased	Manus tissue	COI	LC761143
	KJ-05	Kojima Bay, Okayama	Unknown	Purchased	Manus tissue	COI	LC761144
	KJ-06	Kojima Bay, Okayama	Unknown	Purchased	Abdomen tissue	COI	LC761145
	KJ-07	Kojima Bay, Okayama	Unknown	Purchased	Abdomen tissue	COI	LC761146
	KJ-08	Kojima Bay, Okayama	Unknown	Purchased	Abdomen tissue	COI	LC761147
	KJ-09	Kojima Bay, Okayama	Unknown	Purchased	Abdomen tissue	COI	LC761148
	KJ-10	Kojima Bay, Okayama	Unknown	Purchased	Abdomen tissue	COI	LC761149
	KJ-11	Kojima Bay, Okayama	Unknown	Purchased	Abdomen tissue	COI	LC761150
	AR-01	Arao, Kumamoto	Unknown	Purchased	Manus tissue	COI	LC761151
	AR-02	Arao, Kumamoto	Unknown	Purchased	Manus tissue	COI	LC761152
	AR-03	Arao, Kumamoto	Unknown	Purchased	Manus tissue	COI	LC761153
	AR-04	Arao, Kumamoto	Unknown	Purchased	Manus tissue	COI	LC761154
**B**	**Sample code, Species**	**Collected location**	**Gene**	**Reffrence**	**Accession No.**		
	SK-01	Seosan, South Korea	COI	Kim et al. (2011)	JF793665.1		
	SK-02	South Korea	COI	Kim et al. (Unpublished)	JX502989.1		
	SK-03	South Korea	COI	Kim et al. (Unpublished)	JX502990.1		
	SK-04	Gyeonggi-do, South Korea	COI	Kim, (Unpublished)	OL876961.1		
	SK-05	Gyeonggi-do, South Korea	COI	Kim, (Unpublished)	OL876962.1		
	SK-06	Gyeonggi-do, South Korea	COI	Kim, (Unpublished)	OL876963.1		
	CH-01	Qingdao, China	COI	Liu et al. (2012)	JN897377.1		
	*Upogebiayokoyai*	Jeju-do, Korea	COI	Yang et al. (2014)	NC_025943.1		
	*Wolffogebiaheterocheir*	India	COI	Rengaiyan et al. (2019)	MN579655.1		

### ﻿DNA extraction, PCR amplification and Sequencing

Less than 10 mg of abdominal tissue was removed from adult samples for DNA analysis. DNA was extracted using the ISOSPIN Tissue DNA Kit (NIPPON GENE, Tokyo, Japan) and stored at -25 °C until use.

Polymerase chain reaction (PCR) was used to amplify the COI region of the mitochondrial DNA. Up to 658 bp from the COI region was amplified using the universal primers LCO1490: 5′-GGTCAACAAATCATAAAGATATTG-3′ and HCO2198: 5′-TAAACTTCAGGGTGACCAAAAAATCA-3′ (Folmer et al. 1994). Each 20 μl PCR reaction consisted of 2.0 μl of extracted DNA (undiluted solution), 0.4 μl of KOD FX (1.0 U/μl) (TOYOBO, Osaka, Japan), 4.0 μl of 2.0 mM of each dNTP, 10 μl of 2× PCR buffer for KOD FX, 2.4 μl Nuclease-free water (Thermo Fisher Scientific, Waltham, MA, USA) and 0.6 μl of each primer (10 pmol/μl). Gene Atlas 322 (ASTEC, Fukuoka, Japan) was used as the thermal cycler for PCR. The PCR reactions consisted of an initial denaturation cycle at 94 °C for 2 min, 40 cycles of 98 °C for 10 s, 40 °C for 30 s, 68 °C for 1 min, and a final cycle at 10 °C for 1 min. PCR products were loaded onto an agarose gel with MIDORI Green Advance Agarose Tablets (Nippon Genetics, Tokyo, Japan) and separated by electrophoresis in 1× TAE buffer. Separated DNA fragments were observed under blue/green LED light. All PCR products were purified using Illustra ExoProStar (GE Healthcare, Chicago, USA) and sequenced with a Big Dye Terminator v.3.1 Cycle Sequencing Kit (Thermo Fisher Scientific, Waltham, MA, USA) by contracting to the FASMAC sequencing service (FASMAC, Kanagawa, Japan).

### ﻿Population and phylogenetic analyses

We conducted sequence analyses on 53 specimens of *U.major* for the COI gene, incorporating data obtained from GenBank for three species: *U.major* from Korea (Accession numbers: JF793665, JX502989, JX502990, OL876961, OL876962, OL876963) and China (JN897377), the closest relative species *U.yokoyai* (NC_025943) and *Wolffogebiaheterocheir* (MN579655) as an outgroup, all with COI gene sequences (Table 1). All sequences were aligned using the MEGA X software (Kumar et al. 2018) and adjusted using the BioEdit v.7.0.5.3 software (http://www.mbio.ncsu.edu/BioEdit/bioedit.html). The assembled sequences were manually inspected for quality. Unique haplotypes were identified and used for further analyses. Obtained haplotypes are deposited via DNA Data Bank of Japan (DDBJ) with DDBJ/EMBL/GenBank accession numbers (LC761102–LC761154). Phylogenetic trees were constructed using the maximum-likelihood (ML) method, based on the Tamura 3-parameter model with a discrete gamma distribution (T92 + G) (Suppl. material 1: appendix S1) and the reliability of the trees was tested with 1,000 bootstrap replicates (Felsenstein 1985) using MEGA X software (Kumar et al. 2018). The MEGA X software was utilised to ascertain the differential count of bases between sequences through the application of the “No. for the difference” metric. The “Kimura 2-parameter model” was used to determine the genetic distance between the sequences.

The estimation of haplotypes, haplotype diversity (*h*) and nucleotide diversity (*π*) was performed through the utilisation of DnaSP v.6.12.03. The *F_ST_* values between populations were executed using Arlequin v.3.5.2.2 and the K2P distance metric (Kimura 1980). A haplotype network was constructed to visually depict the genetic distances between haplotypes using Network 10 (Forster et al. 1996). Furthermore, AMOVA was carried out by utilising Arlequin v.3.5.2.2 to identify any disparities in genetic structure amongst the groups. The haplotype data and the preparation of datasets in .hap and .arp format were performed through the use of DnaSP v.6.10.01. The relevant *p*-values were determined through 1023 permutations.

### ﻿Morphological examination

**Figure 2.**
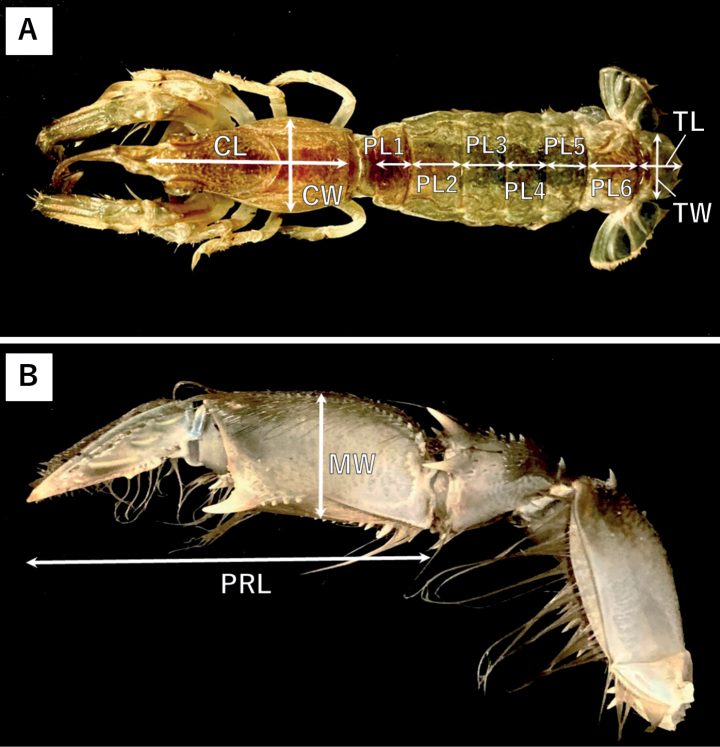
The site at which the morphometric assessments were conducted of *U.major***A**CL: Carapace Length, CW: Carapace Width, PL 1–6: Pleomeres Length, 1–6, PTL: Pleomeres Total Length, TL: Telson Length, TW: Telson Width **B** MW: Manus Width, PRL: Propodus Length.

The parts for morphometric measurements are shown in Fig. 2. An electronic caliper (Shinwa, Niigata, Japan) was employed to ascertain the carapace length and width (CL, CW), as well as the length of the six pleomeres (PL 1 to 6). Additionally, measurements were obtained for the telson length and width (TL, TW). The propodus length (PRL) and the width of the manus (MW) were also determined bilaterally. Furthermore, considering the fact that at least female individuals achieve sexual maturity upon attaining a minimum CL of 25 mm (Kinoshita et al. 2003), we probed the correlation between CL and other morphological parameters in individuals with a CL greater than 25 mm. It is worth noting that the measurement data of adult *U.yokoyai* (n = 8), the closest relative species, were also included in this analysis to examine the inter-species differences in morphology. The specimens of *U.yokoyai* used in this study were collected in Uranouchi Inlet, Kochi, Southwest Japan in October 2021. All measurements were performed to the nearest 0.01 mm. To discriminate variations in morphological characteristics, we performed an extensive observation and analysis of all traits, including the parts where morphometric measurements were taken, by utilising the AndonStar ADSM302 video microscope.

### ﻿Data resources

The data underpinning the analysis reported in this paper are deposited at GBIF, the Global Biodiversity Information Facility and are available at https://doi.org/10.15468/wmdf6k.

## ﻿Results

### ﻿Molecular phylogeny and genetic diversity

The final compilation of data comprised 637 bp of COI sequences from a total of 62 samples of *U.major*, outgroup species *W.heterocheir* and the closely-related outgroup species *U.yokoyai*.

**Figure 3.**
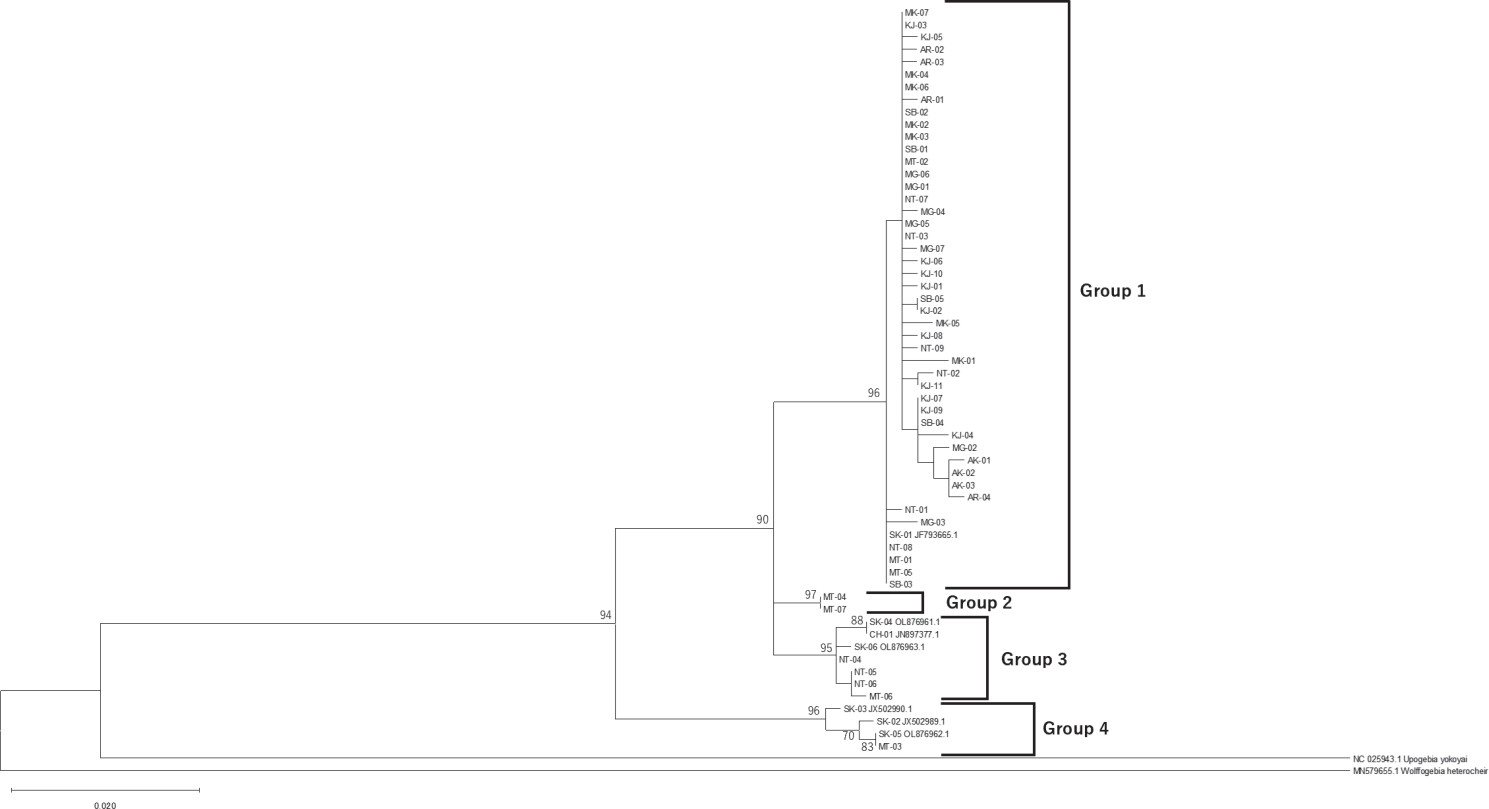
Maximum likelihood tree of concatenated COI sequences. Only bootstrap values exceeding a threshold of 70% were exhibited.

Concatenate sequences are shown in Fig. 3. Based on the ML analyses, *U.major* is distinctly separated from its outgroups and closest species, forming its own clade, in which four groups were recognised. Group 1 constitutes a clade primarily composed of Japanese specimens, with only one individual from South Korea (SK-01) included. Conversely, Group 2 forms a clade exclusively comprised of Matsukawa-ura specimens (MT-04, 07). Group 3 encompasses specimens from Lake Notoro, Matsukawa-ura, Korea and China. Group 4, in contrast, constitutes an independent clade comprised of three specimens from Korea and one from Matsukawa-ura (MT-03). Groups 1 and 2 were of Japanese descent, except for one specimen, whereas Groups 3 and 4 consist of both Japanese and continental specimens forming the clade.

The disparity in base number between Group 2 and Group 3 was found to be minimal, ranging from 7 to 9 bp, while Group 1 and Group 4 showed a significant difference of 29 to 34 bp (Table 2A). Additionally, the genetic distance between Group 2 and Group 3 was small, ranging from 0.011 to 0.014, whereas Group 1 and Group 4 displayed a larger difference, ranging from 0.047 to 0.056 (Table 2B). In particular, MT-03, a specimen of Group 4, exhibited a maximum disparity of 34 bp in base counts and a maximum genetic distance of 0.056 from MT-02, the Matsukawa-ura specimen pertaining to Group 1, thereby rendering them genetically remote from each other. These findings imply that Matsukawa-ura harbours *U.major* with highly divergent genetic characteristics within a single location.

**Table 2.** (A) The maximum, minimum, mean and standard deviation of the base number differences between groups. (B) The maximum, minimum, mean and standard deviation of the genetic distance between groups.

		Between Group 1 and Group 2	Between Group 1 and Group 3	Between Group 1 and Group 4	Between Group 2 and Group 3	Between Group 2 and Group 4	Between Group 3 and Group 4
A	Maximum	14	17	34	9	27	29
	Minimum	10	11	29	7	25	25
	Mean	11.5	13.8	31.3	8.3	26.0	26.8
	Standard deviation	0.862	1.114	1.271	0.699	1.000	1.115
B	Maximum	0.022	0.027	0.056	0.014	0.044	0.047
	Minimum	0.016	0.018	0.047	0.011	0.041	0.041
	Mean	0.018	0.022	0.051	0.013	0.042	0.043
	Standard deviation	0.001	0.002	0.002	0.001	0.002	0.002

**Figure 4.**
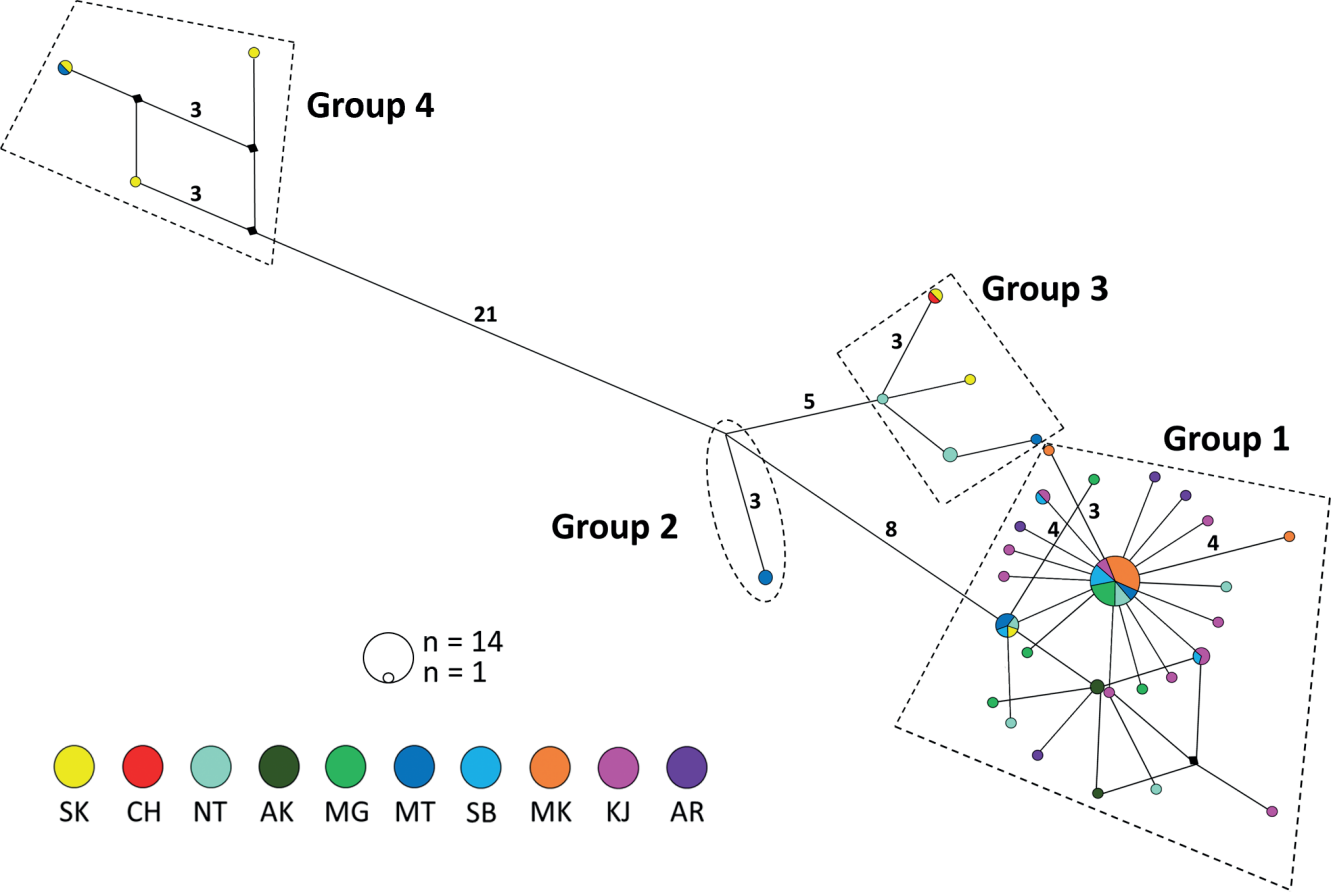
Median-joining haplotype networks, based on COI sequences. The circle size is proportional to the frequency of haplotypes. Black circles represent missing haplotypes. Each line represents a double mutation step, otherwise step numbers are indicated.

Utilisation of the DnaSP software yielded 35 COI haplotypes (Table 3). Lake Notoro and Matsukawa-ura exhibited a predilection for elevated levels of haplotype diversity (*h*: 0.944, 0.905) and nucleotide diversity (*π*: 0.0115, 0.0224), respectively, in comparison to the remaining populations. In contrast, Sanbanze and Kojima Bay displayed higher values of haplotype diversity within the range of 0.900, but comparatively lower base diversity. Haplotype network analysis revealed that the haplotypes of *U.major* could be segregated into four principal groups (Fig. 4), which revealed the genetic distances between the four groups shown in the phylogenetic tree. Moreover, it was explicitly demonstrated that Group 4, which encompasses MT-03, is genetically distant from other groups.

**Table 3.** Frequencies of selected haplotypes, haplotype diversity (*h*) and nucleotide diversity (*π*) in local samples from Japan, South Korea and China.

COI haplotype	Local populations									Total
	SK/CH	NT	AK	MG	MT	SB	MK	KJ	AR	
H_1	1	0	0	0	0	0	0	0	0	1
H_2	1	0	0	0	0	0	0	0	0	1
H_3	1	0	0	0	0	0	0	0	0	1
H_4	1	0	0	0	1	0	0	0	0	2
H_5	2	0	0	0	0	0	0	0	0	2
H_6	1	1	0	0	2	1	0	0	0	5
H_7	0	0	1	0	0	0	0	0	0	1
H_8	0	0	2	0	0	0	0	0	0	2
H_9	0	1	0	0	0	0	0	0	0	1
H_10	0	1	0	0	0	0	0	0	0	1
H_11	0	2	0	3	1	2	5	1	0	14
H_12	0	1	0	0	0	0	0	0	0	1
H_13	0	2	0	0	0	0	0	0	0	2
H_14	0	1	0	0	0	0	0	0	0	1
H_15	0	0	0	0	0	0	0	0	1	1
H_16	0	0	0	0	0	0	0	0	1	1
H_17	0	0	0	0	0	0	0	0	1	1
H_18	0	0	0	0	0	0	0	0	1	1
H_19	0	0	0	0	2	0	0	0	0	2
H_20	0	0	0	0	1	0	0	0	0	1
H_21	0	0	0	0	0	0	1	0	0	1
H_22	0	0	0	0	0	0	1	0	0	1
H_23	0	0	0	0	0	0	0	1	0	1
H_24	0	0	0	0	0	1	0	1	0	2
H_25	0	0	0	0	0	0	0	1	0	1
H_26	0	0	0	0	0	0	0	1	0	1
H_27	0	0	0	0	0	0	0	1	0	1
H_28	0	0	0	0	0	1	0	2	0	3
H_29	0	0	0	0	0	0	0	1	0	1
H_30	0	0	0	0	0	0	0	1	0	1
H_31	0	0	0	0	0	0	0	1	0	1
H_32	0	0	0	1	0	0	0	0	0	1
H_33	0	0	0	1	0	0	0	0	0	1
H_34	0	0	0	1	0	0	0	0	0	1
H_35	0	0	0	1	0	0	0	0	0	1
*h*	0.952	0.944	0.667	0.857	0.905	0.900	0.524	0.982	1.000	60
*π*	0.02841	0.01151	0.00105	0.00344	0.02235	0.00188	0.00224	0.00320	0.00471	

Pairwise *F_ST_* revealed genetic differentiation in all groups (*F_ST_* = 0.7931 to 0.9388, *p* < 0.05) (Table 4). The *F_ST_* values between Groups 1 and 2, primarily comprising Japanese specimens, and Group 3, consisting of continental specimens, were 0.8445 and 0.7931, respectively. Group 2 demonstrated substantial genetic divergence from both groups, although it was only marginally closely related to Group 3. The most notable genetic difference was observed between the Group 1 and 4 populations, with a recorded *F_ST_* value of 0.9388.

**Table 4.** Pairwise *F_ST_* values amongst groups.

	Group 1	Group 2	Group 3	Group 4
Group 1	0.0000			
Group 2	0.8445	0.0000		
Group 3	0.8608	0.7931	0.0000	
Group 4	0.9388	0.9256	0.9177	0.0000

A quantitative assessment of the genetic structural disparities between each group was conducted using AMOVA (Table 5). The analysis showed significant differences in the genetic structure between the groups, with most genetic differentiation occurring amongst the groups (86.97%, *F_ST_* = 0.900). Conversely, the variation within each group was observed to be quite minimal, measuring at a mere 9.20%.

**Table 5.** AMOVA for COI sequences of *U.major* populations used in this study. *d.f.* indicates degrees of freedom. * indicates *p* < 0.01.

Source of variation	*d.f.*	Sum of squares	% of variation	Fixation index
Among Groups	3	197.197	86.97	*F_ST_* = 0.900*
Within Groups	56	54.772	9.20	

### ﻿Morphological characteristics

**Figure 5.**
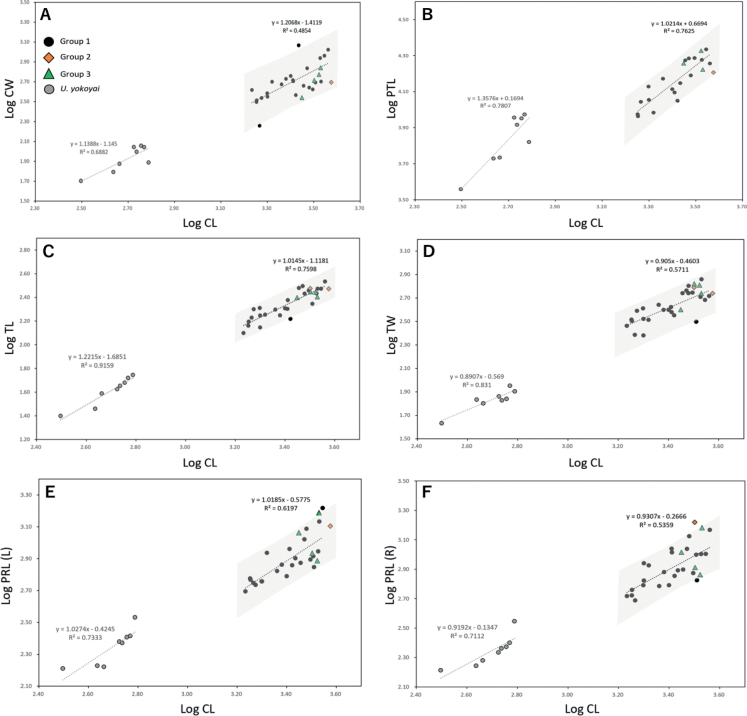
The size of each of the morphological parameters relative to carapace length (CL) **A** Carapace Width (CW) **B** Pleomere Total Length (PTL) **C** Telson Length (TL) **D** Telson Width (TW) **E** Left Propodus Length (PRL (L)), and **F** Right Propodus Length (PRL (R)). Shaded areas show the 95% confidence interval. Note that Group 4 was excluded from the analysis due to the carapace of the MT-03 being damaged, rendering the measurement of CL impossible.

**Figure 6.**
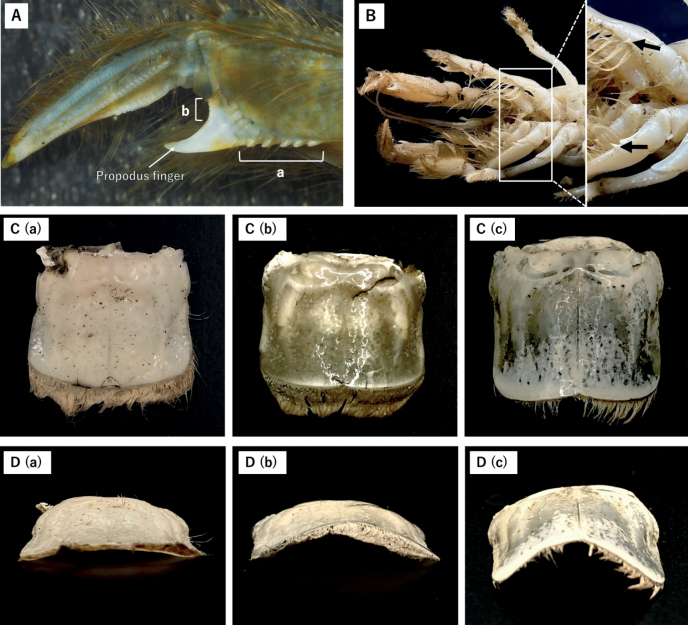
Morphological traits observed in this study **A** projections located beneath the manus (a) and above the propodal finger (b) **B** ventral projections of pereopod 2 (arrow) **C** telson tip. (a) Linear shape, Female, MT-01 (b) Slightly concave shape, Male, MT-02 (c) Concave shape, MT-03 specimen from Matsukawa-ura **D** cross-sectional profile of the telson. (a) Linear shape, Female, MT-01 (b) Slightly arched shape, Male, MT-02 (c) Arched shape, MT-03

All measures of morphological traits of Japanese *U.major* are presented in Table 6 (see Suppl. material 1: appendix S4 for data distinguished by sex). The data gathered were evaluated for size disparities amongst groups and genders using the Tukey test. The results showed statistically significant differences only in the MW (right) size between the genders, while no other significant differences were observed (refer to Suppl. material 1: appendix S5). It should be noted that the between-group testing was restricted to Group 1 versus Group 3, considering the limited sample sizes of Groups 2 and 4 (n = 1–2). Furthermore, the relationship between CL and each morphological parameter was analysed to determine variations amongst groups, as shown in Fig. 5 (see also Suppl. material 1: appendix S6). By utilising Group 1 as the benchmark, which possesses the largest sample size in this study, and establishing 95% confidence intervals, the male samples originating from Groups 2 and 3 exhibited marginally higher PRL/CL ratios than those from Group 1 (Fig. 5E, F and Suppl. material 1: appendix S6E (a), see F (a) for more detail). Additionally, the F-test conducted on the slopes of the data for Group 1 and *U.yokoyai* did not reveal any statistically significant differences, except for PTL (Male) (Suppl. material 1: appendix S7). This suggests that the two species share similar growth patterns, but exhibit distinct size variations. However, due to the small sample size, the statistical analysis of the slope was not performed for all groups except Group 1. Therefore, it cannot be ruled out that the other groups may have different slopes for Group 1, indicating that Groups 2 and 3 may display divergent growth patterns from Group 1. To investigate this possibility, it would be necessary to augment the sample size in each group. On the other hand, the analysis of morphological features involved quantification of the number of projections located below the manus (NPLBM) and the number of projections above the propodal finger (NPAPF) and tabulation of the number of ventral projections of pereopod 2 (NVPP2) (Fig. 6A, B). Note that this analysis specifically focused on specimens possessing a CL of 25 mm or larger. The results demonstrate that NPLBM, NPAPF and NVPP2 had a range of 3 to 7, 2 to 9 and 1 to 3, respectively, in Group 1 of Japanese descent, while NPLBM ranged from 4 to 7, NPAPF from 6 to 9 and NVPP2 from 1 to 2 in the continental Group 3 (Table 7). Moreover, amongst the parameters, NPAPF was the only one that exhibited a significant difference between males and females, with a range of 3 to 9 in males and 2 to 8 in females (see Suppl. material 1: appendix S8). Despite the variability in the number of projections, the Tukey test revealed no statistically significant differences in any of the parameters between groups or genders (Suppl. material 1: appendix S9). By contrast, there were noticeable disparities in the morphology of the telson apices between the sexes (Suppl. material 1: appendix S8). In males, approximately 90% displayed a concave shape in the median, whereas all females demonstrated a linear shape (Fig. 6C). Amongst the specimens, one stands out as particularly distinctive: MT-03, a male individual from Group 4, which exhibits a conspicuously concave-shaped tip, a trait that is absent in all other specimens. Additionally, the majority of the talus cross-sectional shapes were linear, exceeding 90% in both groups and genders, with only a minority of specimens displaying a slightly arched talus. In contrast, MT-03 presented a marked arch shape, providing a distinct contrast from the other specimens (Fig. 6D).

**Table 6.** The morphometric measurements’ outcomes have been tabulated by group. The maximum and minimum values and the corresponding standard deviation, have been presented for each Group, except for group 4 (n = 1). The symbol “-” denotes that the observation was unattainable due to impairment.

Group	Sample Code	Sex	CL	CW	PL1	PL2	PL3	PL4	PL5	PL6	PTL	TL	TW	MW		PRL	
														Left	Right	Left	Right
1	NT-01	Male	32.46	14.06	10.17	-	9.05	8.70	9.58	11.01	-	-	16.51	7.96	7.88	21.92	22.74
	NT-02	Male	34.17	14.92	10.49	14.89	9.93	-	10.25	11.64	-	11.85	17.48	8.26	-	22.93	-
	NT-03	Male	-	-	11.94	8.71	-	-	8.06	11.13	-	10.48	14.13	8.33	8.31	23.40	8.31
	NT-07	Female	31.69	14.31	11.57	11.10	9.28	8.94	10.68	11.23	71.74	11.93	15.53	5.11	4.95	17.70	18.13
	NT-08	Female	32.97	13.79	10.60	12.61	10.13	9.44	9.05	11.39	72.66	11.68	15.60	5.79	5.22	18.07	17.71
	NT-09	Female	33.36	14.79	11.49	11.69	9.95	10.92	10.74	-	-	-	-	5.47	6.33	18.45	20.04
	AK-01	Female	33.46	-	-	-	-	8.63	7.20	10.25	-	10.44	12.17	5.56	5.24	17.23	16.85
	AK-02	Female	-	-	-	-	-	-	-	-	-	-	-	7.87	-	23.67	-
	AK-03	Female	-	-	-	-	-	-	-	-	-	-	-	-	8.24	-	24.48
	MG-01	Female	26.43	12.65	9.38	11.07	-	8.27	9.07	10.68	-	9.97	13.35	5.00	-	15.41	-
	MG-02	Male	31.03	21.49	10.21	11.17	8.19	7.68	8.70	9.61	63.24	-	-	6.06	6.22	18.22	18.03
	MG-03	Female	-	-	11.15	12.58	10.35	9.57	-	12.51	65.73	11.51	14.76	-	-	-	-
	MG-04	Female	19.26	8.57	6.10	-	-	-	5.86	7.49	-	7.02	8.60	3.24	3.12	10.81	10.77
	MG-05	Female	18.81	11.07	6.34	-	5.34	5.17	5.68	7.79	-	6.71	7.76	3.42	3.39	11.04	11.60
	MG-06	Male	21.95	8.73	6.87	8.09	-	-	5.97	7.51	-	6.71	9.83	4.10	3.72	13.23	12.52
	MG-07	Male	30.32	14.99	9.87	-	8.51	7.72	8.60	9.66	-	10.77	13.21	6.62	7.02	19.31	20.38
	MT-01	Female	32.44	-	10.13	12.63	9.13	7.58	7.46	11.49	66.00	11.38	15.51	-	-	-	-
	MT-02	Male	26.20	9.59	8.31	11.23	6.98	6.90	7.56	9.12	57.00	9.29	10.88	4.67	4.54	15.61	14.69
	MT-05	Male	-	-	-	-	-	-	-	-	-	11.49	15.33	-	8.89	-	23.64
	SB-01	Male	27.12	-	8.70	9.87	6.67	6.98	9.18	9.27	57.65	9.45	12.47	-	5.58	-	18.92
	SB-02	Female	27.08	13.28	9.87	11.50	8.76	6.82	8.81	9.47	62.05	10.07	13.64	4.67	4.97	15.75	16.30
	SB-03	Male	25.37	13.72	6.52	8.85	-	6.02	7.53	8.22	-	8.15	11.75	5.24	5.39	14.79	15.14
	SB-04	Female	-	-	9.18	10.43	-	-	-	-	-	-	-	5.14	-	16.09	-
	SB-05	Male	29.38	15.34	-	-	-	-	-	9.45	-	9.46	13.46	5.71	6.34	17.49	17.83
	MK-01	Male	25.89	12.36	8.68	10.14	6.85	5.99	6.40	8.57	52.62	8.98	12.24	5.09	4.81	15.93	15.78
	MK-02	Male	30.27	15.14	9.43	10.72	7.56	7.20	8.34	9.56	60.01	10.00	13.79	-	7.17	-	20.88
	MK-03	Male	30.64	13.03	9.29	10.38	7.60	6.96	7.53	8.66	57.38	9.19	12.85	6.08	6.00	17.44	17.36
	MK-04	Male	27.66	14.91	9.14	9.52	6.50	6.45	6.82	8.81	53.69	9.52	12.37	6.03	6.15	18.84	18.65
	MK-05	Male	23.85	11.81	8.01	8.49	5.82	6.68	7.30	7.40	50.38	8.37	11.08	4.74	4.82	14.47	15.39
	MK-06	Female	28.82	14.53	9.75	11.36	8.19	8.36	9.12	9.64	64.78	9.94	14.05	5.16	5.05	16.81	16.21
	MK-07	Female	29.97	15.79	9.58	10.91	7.30	7.39	8.79	9.78	61.14	10.05	13.46	4.91	5.38	16.28	16.30
	KJ-01	Female	23.99	12.76	8.30	9.51	6.25	6.10	6.43	8.13	50.82	8.52	11.46	5.57	5.66	16.09	17.35
	KJ-02	Female	22.16	10.00	6.93	6.84	5.69	4.72	5.63	7.05	41.58	7.19	9.76	3.98	4.36	12.05	13.10
	KJ-03	Female	23.34	10.59	7.42	8.63	6.18	6.13	6.46	7.77	48.72	7.65	11.01	4.36	4.33	13.25	13.30
	KJ-04	Male	25.86	12.17	8.03	9.56	6.94	6.67	7.31	8.03	53.21	8.68	12.39	5.32	5.03	16.06	15.21
	KJ-05	Male	20.88	11.04	7.08	8.21	5.63	5.38	6.02	7.08	44.78	6.96	9.65	4.62	4.61	13.99	13.53
	KJ-06	Female	19.61	10.56	6.93	8.06	5.07	-	5.82	6.78	-	6.98	9.61	4.13	4.05	12.62	12.66
	KJ-07	Male	27.12	12.82	8.53	10.12	6.65	-	7.13	8.05	-	8.55	10.83	5.28	5.89	15.75	16.82
	KJ-08	Female	22.82	11.21	7.36	8.63	6.04	4.67	7.00	7.57	45.94	7.66	10.99	4.71	4.65	13.98	13.70
	KJ-09	Female	22.42	10.01	7.91	9.01	6.20	6.03	6.50	7.58	49.26	7.92	10.27	4.90	4.78	14.16	14.33
1	KJ-10	Male	22.16	10.69	6.74	8.62	5.97	5.88	6.42	7.39	46.90	7.90	10.17	4.81	5.09	14.52	14.33
	KJ-11	Female	23.85	11.17	7.41	9.41	6.15	6.94	7.10	7.37	51.32	7.82	10.18	4.61	4.71	15.31	14.67
	AR-01	Male	35.22	20.57	10.58	11.36	9.24	8.72	10.39	11.53	70.54	12.60	15.14	-	9.95	-	23.75
	AR-02	Female	32.18	17.08	11.50	12.10	10.03	8.84	10.35	10.86	72.52	12.12	15.88	6.73	6.41	20.51	20.85
	AR-03	Female	34.03	18.91	12.42	11.84	9.16	8.62	9.97	11.33	71.96	11.39	15.03	6.22	6.40	19.02	20.14
	AR-04	Female	34.66	19.36	12.19	12.52	9.80	9.83	10.29	11.82	76.28	11.86	14.65	11.48	6.64	24.97	20.17
	Maximum		35.22	21.49	12.42	14.89	10.35	10.92	10.74	12.51	76.28	12.60	17.48	11.48	9.95	24.97	24.48
	Minimum		18.81	8.57	6.10	6.84	5.07	4.67	5.63	6.78	41.58	6.71	7.76	3.24	3.12	10.81	8.31
	Mean		27.5	13.5	9.1	10.3	7.6	7.3	7.9	9.3	58.6	9.5	12.7	5.6	5.7	16.7	16.8
	Standard deviation		4.717	3.093	1.739	1.673	1.608	1.493	1.554	1.620	9.675	1.722	2.316	1.523	1.464	3.362	3.638
2	MT-04	Male	33.17	-	11.89	13.14	8.60	8.15	-	11.24	-	11.89	16.27	-	9.29	-	25.02
	MT-07	Male	35.72	14.80	11.31	11.15	9.14	8.22	8.88	10.21	67.13	11.86	15.49	7.94	-	22.30	-
	Maximum		35.72	14.80	11.89	13.14	9.14	8.22	8.88	11.24	67.13	11.89	16.27	7.94	9.29	22.30	25.02
	Minimum		33.17	14.80	11.31	11.15	8.60	8.15	8.88	10.21	67.13	11.86	15.49	7.94	9.29	22.30	25.02
	Mean		34.4	14.8	11.6	12.1	8.9	8.2	8.9	10.7	67.1	11.9	15.9	7.9	9.3	22.3	25.02
	Standard deviation		1.275	0.000	0.290	0.995	0.270	0.035	0.000	0.515	0.000	0.015	0.390	0.000	0.000	0.000	0.000
3	NT-04	Female	33.89	16.03	11.71	11.12	10.43	9.56	11.70	11.69	75.77	11.53	16.61	5.92	5.58	17.94	17.52
	NT-05	Male	31.46	12.69	11.36	11.03	8.42	9.18	9.76	11.70	70.63	11.01	15.49	7.34	7.14	21.38	20.40
	NT-06	Female	33.20	15.12	-	12.05	9.70	9.66	10.74	12.36	-	11.56	16.80	5.24	5.26	18.83	18.39
	MT-06	Male	34.13	17.16	10.19	12.12	8.82	8.26	9.87	10.70	68.22	11.07	15.49	8.16	7.96	24.26	24.10
	Maximum		34.13	17.16	11.71	12.12	10.43	9.66	11.70	12.36	75.77	11.56	16.80	8.16	7.96	24.26	24.10
	Minimum		31.46	12.69	10.19	11.03	8.42	8.26	9.76	10.70	68.22	11.01	15.49	5.24	5.26	17.94	17.52
	Mean		33.2	15.3	11.1	11.6	9.3	9.2	10.5	11.6	71.5	11.3	16.1	6.7	6.5	20.6	20.1
	Standard deviation		1.045	1.645	0.650	0.507	0.780	0.552	0.781	0.593	3.149	0.254	0.611	1.149	1.110	2.460	2.533
4	MT-03	Male	-	-	-	11.21	9.33	8.26	8.49	11.96	-	11.58	15.63	-	7.39	-	23.68

## ﻿Discussion

The COI phylogenetic tree analysis reveals that *U.major* is distinctly separated from its outgroups and closest species, forming its own clade comprising four groups (Fig. 3). Group 1 is predominantly made up of Japanese specimens, while Groups 3 and 4 consist of continental specimens, categorising them broadly as Japanese and continental, respectively. However, Group 2 specimens from Matsukawa-ura exhibit genetic differentiation from both continental and Japanese descent and the presence of Japanese individuals from Matsukawa-ura and Lake Notoro in Groups 3 and 4, along with significantly larger PRL of Group 2 and 3 individuals of males in comparison to Group 1, suggest that genetically and morphologically distinct *U.major* is prevalent in Japan.

**Table 7.** The results of morphometric observations are summarised by group. NPLBM: Number of projections located beneath the manus, NPAPF: Number of projections above the propodal finger, NVPP2: Number of ventral projections of pereopod 2, MTT: Morphology of the telson tip, CST: Cross-sectional shape of the telson, L: Linear, SC: Slightly concave, C: Concave, SA: Slightly arched, A: Arched. The maximum and minimum values and the corresponding standard deviations are given for each group, except for Group 4, where the sample size is 1. Additionally, the percentage of MTT and CST traits are presented. The symbol “-” indicates an unattainable observation due to impairment.

Group	Sample code	Sex	NPLBM		NPAPF		NVPP2			MTT	CST
			Left	Right	Left	Right	Left	Right			
1	NT-01	Male	5	4	8	8	–	1		SC	L
	NT-02	Male	5	–	6	–	2	1		SC	L
	NT-07	Female	4	5	4	7	2	2		L	L
	NT-08	Female	6	5	7	4	1	1		L	L
	NT-09	Female	5	4	–	2	–	1		L	L
	AK-01	Female	5	6	5	4	1	1		L	L
	MG-01	Female	4	–	–	–	–	2		L	L
	MG-02	Male	3	4	8	9	3	2		SC	L
	MG-07	Male	5	4	5	6	2	2		L	L
	MT-01	Female	–	–	–	–	–	1		L	L
	MT-02	Male	4	5	4	6	2	2		SC	SA
	SB-01	Male	–	5	–	6	2	–		SC	L
	SB-02	Female	–	3	–	8	2	1		L	L
	SB-03	Male	5	6	4	5	2	1		SC	L
	SB-05	Male	6	5	4	6	2	2		SC	L
	MK-01	Male	7	6	4	3	2	2		SC	L
	MK-02	Male	–	4	–	6	2	2		SC	L
	MK-03	Male	6	6	6	5	2	2		SC	L
	MK-04	Male	5	5	7	5	2	2		SC	L
	MK-06	Female	4	4	6	4	2	2		L	L
	MK-07	Female	5	6	4	6	3	2		L	L
	KJ-04	Male	6	5	5	5	–	–		SC	L
	KJ-05	Male	4	4	7	5	2	2		SC	L
	AR-01	Male	–	5	–	6	2	1		SC	L
	AR-02	Female	4	5	5	2	2	2		L	L
	AR-03	Female	4	4	5	4	2	2		L	L
	AR-04	Female	5	4	5	5	2	2		L	L
	Maximum		7	6	8	9	3	2	%	L = 48.1	L = 96.3
	Minimum		3	3	4	2	1	1		SC = 51.9	SA = 3.7
	Mean		4.9	4.8	5.5	5.3	2.0	1.6		C = 0.0	A = 0.0
	Standard deviation		0.919	0.829	1.322	1.695	0.426	0.480			
2	MT-04	Male	–	6	–	8	1	–		SC	L
	MT-07	Male	4	–	8	–	–	2		SC	L
	Maximum		4	6	8	8	1	2	%	L = 100	L = 100
	Minimum		4	6	8	8	1	2		SC = 0.0	SA = 0.0
	Mean		4.0	6.0	8.0	8.0	1.0	2.0		C = 0.0	A = 0.0
	Standard deviation		0.000	0.000	0.000	0.000	0.000	0.000			
3	NT-04	Female	7	6	–	–	1	2		L	L
	NT-05	Male	4	5	8	7	2	1		SC	L
	NT-06	Female	5	4	6	6	2	2		L	L
	MT-06	Male	5	4	8	9	2	1		SC	L
	Maximum		7	6	8	9	2	2	%	L = 50.0	L = 100
	Minimum		4	4	6	6	1	1		SC = 50.0	SA = 0.0
	Mean		5.3	4.8	7.3	7.3	1.8	1.5		C = 0.0	A = 0.0
	Standard deviation		1.090	0.829	0.943	1.247	0.433	0.500			
4	MT-03	Male	–	4	–	9	–	–		C	A

Although the precise origin of *U.major* remains unknown, the oldest lineage traced in the inferred phylogenetic tree belongs to Group 4, with the South Korean sample (SK-03) being the first to diverge within this group. Thus, it is plausible that the coastal waters of Korea could serve as the putative origin of *U.major*. This study assumes that the origin of *U.major* is situated around South Korea and conducts an extensive evaluation of the genetic differentiation of this species throughout the entirety of the Japanese archipelago, taking into account various physical and anthropogenic factors.

### ﻿The dispersion of genes in marine benthos influenced by physical factors

**Figure 7.**
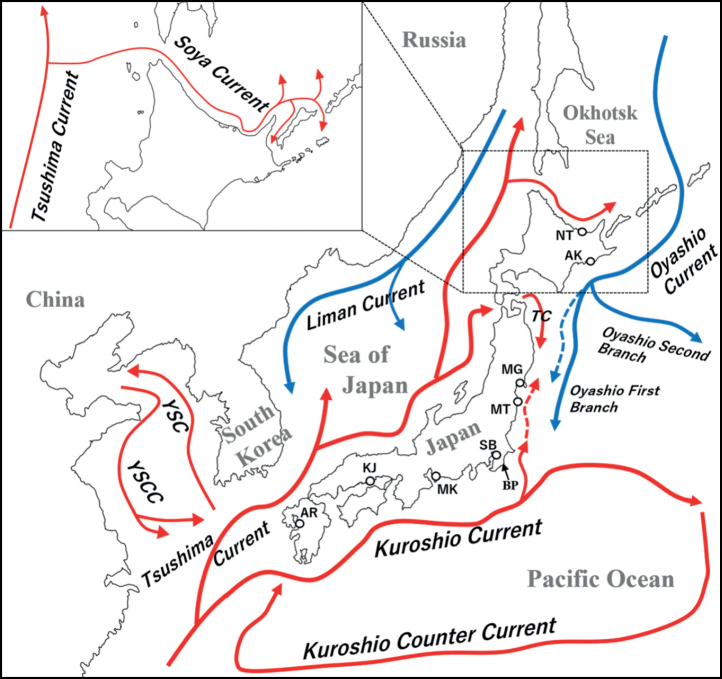
Designations of the oceanic currents and their respective flow patterns in the Japanese archipelago. The Blue dotted arrow denotes the southern extremity of the First branch of the Oyashio current during its attenuation, while the Red dotted arrow demarcates the northern boundary of the Kuroshio Basin at that particular juncture. TC: Tsugaru Current, YSC: Yellow Sea Current, YSCC: Yellow Sea Counter Current, BP: Boso Peninsula.

Marine benthos possessing planktonic larval stages have the potential to increase their geographic range owing to their floating period and the hydrodynamic properties of ocean currents. Specifically, around the Japanese archipelago, three prominent oceanic currents exist: the warm Tsushima Current, the Kuroshio Current and the cold Oyashio Current (Fig. 7). According to the Japan Meteorological Agency, the Kuroshio Current, which flows eastwards along the Pacific coast of the Japanese archipelago, usually reaches the Boso Peninsula, where Sanbanze is located. However, when the first branch of the Oyashio Current, flowing southwards from the north, is weakened, the Kuroshio Current extends northwards off the coast of Miyagi Prefecture, where Mangoku-ura is located. Additionally, the Kuroshio Current which flows offshore of the Boso Peninsula into the Pacific Ocean, voyages towards the south along the southern extent of the Japanese archipelago as the Kuroshio Counter Current, meandering westwards and ultimately reunites with the principal Kuroshio Current. The Tsushima Current, a warm current, flows through the Sea of Japan, and a segment thereof permeates the Pacific Ocean as the Tsugaru Current. The Soya Current is the constituent that transits through the Soya Strait and infiltrates the Sea of Okhotsk.

Thus, ocean currents circulate around the Japanese archipelago. *U.major* has three larval stages and one decapod stage and its planktonic larval period is estimated to be approximately one month or more for individuals inhabiting Tokyo Bay (Kinoshita et al. 2003). The flow velocity of major ocean currents near Japan varies seasonally, but generally, the Kuroshio flows at 2 to 3 knots (3.7 to 5.6 km/h), the Tsushima Current at 1 to 1.5 knots (1.9 to 2.8 km/h) and the Oyashio at approximately 1 knot (Japan Coast Guard 2023). Assuming a planktonic larval period of 31 d for *U.major*, maximum displacement distances of up to 4,166 km in the Kuroshio region, 2,083 km in the Tsushima Current region and 1,414 km in the Oyashio region are possible.

**Figure 8.**
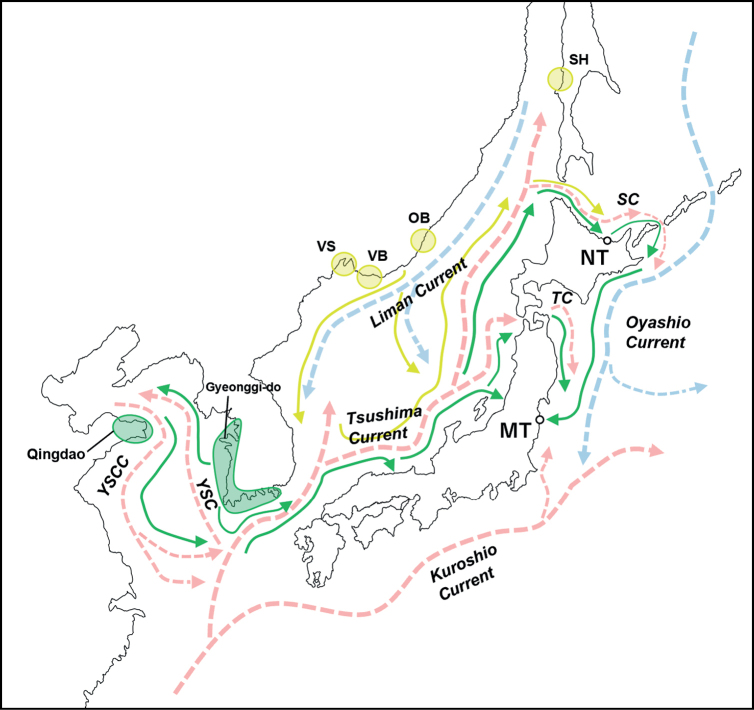
Possible routes for *U.major* larvae from South Korea, China and Russia to Lake Notoro and Matsukawa-ura. The warm currents are denoted by red dotted arrows, while the cold currents are denoted by blue dotted arrows. The collection sites of specimens from Korea and China used in this study are indicated by green circles, while the localities of *U.major* in Russia are denoted by yellow circles (The exact collection site in Sakhalin Island is unknown). Furthermore, potential dispersal routes of *U.major* larvae from South Korean/Chinese specimens and Russian specimens are shown in green and yellow arrows, respectively. SC: Soya Current, TC: Tsugaru Current, YSC: Yellow Sea Current, YSCC: Yellow Sea Counter Current, VS: Vladivostok, VB: Vostok Bay, OB: Olga Bay, SH: Sakhalin Island.

The presence of *U.major* individuals belonging to Groups 3 and 4 in Lake Notoro and Matsukawa-ura, respectively, implies the potential for passive continental invasion of the species into Japan through the agency of oceanic currents. Included in Groups 3 and 4 were specimens from Gyeonggi-do, a coastal region situated in the western part of South Korea, as well as specimens from the Qingdao Peninsula in China and South Korean specimens with unspecified collection locations. To reach Lake Notoro, these individuals must follow the Yellow Sea Coastal Current and subsequently merge with the Tsushima Current before finally riding along the Soya Current (Fig. 8). A similar example of marine organisms expanding their range through such mechanisms is demonstrated by *Nemopilemanomurai*. This species is known to inhabit the Bohai, Yellow and East China Seas before infiltrating the Sea of Japan via the Tsushima Current (Uye 2008; Moon et al. 2010). Therefore, it is possible that planktonic larvae of continental *U.major* might have traversed the Sea of Japan through this route. Nevertheless, the velocity of ocean currents and the duration of *U.major* planktonic larvae render direct transport from the Yellow Sea arduous, which indicates the possibility of an intermediate relay point on the Sea of Japan side of the Japanese archipelago. Additionally, this species has been sighted in several regions of Russia, including Vladivostok, Vostok Bay, Olga Bay and Sakhalin Island, but without any corresponding genetic data (Makarov 1938; Urita 1942; Vinogradov 1950; Selin 2017).

While no prior research has documented the transfer of organisms from the Russian coastline to the Japanese archipelago via the Liman Current merging with the Tsushima Current, it is possible that *U.major* larvae from Russia may have travelled this route and ultimately arrived at Lake Notoro. Moreover, it is worth noting that although a greater distance must be traversed to reach Matsukawa-ura, two potential routes may be posited: one involves planktonic larvae of continental *U.major* inhabiting Lake Notoro riding the Soya Current into the Pacific Ocean and then utilising the Oyashio Current to reach Matsukawa-ura and the other involves reaching Matsukawa-ura via the Tsugaru Current from an intermediate relay point on the Sea of Japan (Fig. 8).

While the sample size is not extensive, the evident genetic and morphological heterogeneity exhibited by the *U.major* population in Matsukawa-ura is noteworthy. This diversity may, to some extent, be influenced by changes in coastal topography. The Tohoku region, where Matsukawa-ura is situated, experiences significant earthquakes once every 500–800 years (Sawai et al. 2012), which can trigger tsunamis leading to alterations in coastal topography. The most recent such occurrence was the Great East Japan Earthquake in March 2011, which resulted in the destruction of the sandbar in Matsukawa-ura and allowed coastal water to inundate the area, significantly modifying the topography (Nishi et al. 2012). However, such disturbances can also increase species diversity. For example, after the Great East Japan Earthquake, the range of *Gasterosteus* genera in the Tohoku region expanded due to changes in topography, resulting in hybridisation and increased genetic and morphological diversity (Hosoki et al. 2019). Additionally, the distribution range of oysters expanded as a result of wide-scale transport of oyster reefs by tsunamis generated during the earthquake (Okoshi 2016). There have been no reports indicating changes in the distribution range of *U.major* after the Great East Japan Earthquake. There have been no reports indicating changes in the distribution range of *U.major* after the Great East Japan Earthquake. However, there is a possibility that the alterations in coastal topography caused by the earthquake and tsunami, along with the influx of coastal water, may have advanced genetic differentiation.

Additionally, an example of the spread of organisms of continental origin across the Japanese archipelago is evident in the “continental relict species”. During the Pleistocene glacial period, the southern coast of Korea and the present-day Ariake Sea were united by land, but regression of the Ariake Sea during the postglacial period resulted in their separation (Emery et al. 1971; Wang and Wang 1980). Consequently, the habitats of living beings have also separated and, today, the Ariake Sea is a habitat for many benthic organisms that possess continental genes (Sato 2010). However, the distribution of many continental relict species in the Ariake Sea has not expanded. This could be due to the difficulty of maintaining a shared inner bay environment with continental coasts in Japan, which are isolated from the continent (Sato 2010). Initially, it remains uncertain whether *U.major* was extant during the time in question, given the lack of fossil evidence. Nevertheless, based on current perspectives, it appears somewhat improbable that this species is a relict of a continental nature. Furthermore, all specimens from the Ariake Sea are included in Group 1 and their genetic relationships with continental species cannot be determined. To evaluate this hypothesis, more comprehensive examinations of Ariake Sea specimens are required, followed by an estimation of their divergence ages and other relevant details.

Group 1 individuals shared the haplotype H_11 at six different sites in Japan (Lake Notoro, Mangoku-ura, Matsukawa-ura, Sanbanze, Mikawa Bay and Kojima Bay) and displayed a trend of low genetic diversity, indicating that their planktonic larvae were dispersed over a broad north-south range by ocean currents surrounding the Japanese archipelago. *Panulirusjaponicus*, a decapod crustacean resembling *U.major*, is broadly distributed in the Kuroshio region, but there is no identifiable genetic variation within populations. With a planktonic larval duration of about one year, it is speculated that *P.japonicus* with the same haplotype has moved long distances over an extended period through the Kuroshio region (Inoue et al. 2007). *Macrophthalmusjaponicus*, extensively distributed across the intertidal zones of Japan, possesses an extended planktonic larval phase exceeding one month, enabling extensive dispersal throughout the region through the influence of the Kuroshio Current. As a consequence, this species demonstrates limited genetic diversity and lacks clear population structuring throughout the Japanese archipelago (Kobayashi et al. 2023). Moreover, marine gastropods of the genus *Monodonta*, also widely distributed in Japan, exhibit a briefer planktonic larval period of approximately 3 to 5 days; however, they reveal genetically distinct populations broadly distributed between the Japan Sea side and the Pacific side, facilitated by dispersal via the Tsushima Current and the Kuroshio Current (Yamazaki et al. 2017). Therefore, it is possible that *U.major* also follows a similar dispersal pattern. The dispersal range of marine benthic larvae is affected by a variety of environmental factors, including water temperature, pH, salinity, dissolved oxygen concentration, ultraviolet radiation and turbidity, as well as biological factors, such as the availability of food and the presence of predators and habitat preferences (Cowen and Sponaugle 2009; Yamazaki et al. 2020; Bashevkin et al. 2020). Thus, it is improbable that the larvae of *U.major* dispersed passively solely through the influence of ocean currents; however, it cannot be excluded that they may have disseminated throughout the entire coastal area of Japan by utilising ocean currents for transportation.

In contrast, two specimens (MT-04 and 07) from Matsukawa-ura, belonging to Group 2, exhibited a slight difference in PRL compared to Group 1 and no discernible morphological distinctions were noted between Group 2 and Group 3. Nevertheless, they exhibited a distinctive haplotype, which may suggest phenotypic plasticity. This occurrence is well established in marine snails, as demonstrated in previous studies (e.g. Hollander et al. (2006); Kurihara et al. (2006)). Furthermore, Matsukawa-ura was found to harbor individuals from all four groups, but it is plausible that the specimens belonging to Group 2 may have migrated from collection sites not surveyed in this study. Alternatively, the individuals in Group 2 may have differentiated within Matsukawa-ura, with the subsequent movement of individuals from Groups 1, 3 and 4 to this location. To determine the exact distribution range of Group 2, a more extensive collection survey is necessary.

### ﻿The dispersion of species as a consequence of anthropogenic activities

In addition to physical factors, it is possible that species dispersion occurs because of anthropogenic activities. Japan began importing the Manila clam *Ruditapesphilippinarum*, from China and North Korea in the 1980s. Jute bags of imported clams contain live organisms other than clams, which are released into domestic clam fisheries or added to aquaculture farms every year (Okoshi 2004; Okoshi and Sato-Okoshi 2011). One species, the moon snail *Lagunculapulchella*, is established throughout Japan and is a significant predator of clams. Additionally, crustacean species, such as *Philyrapisum* and *Pagurus* sp., have also been found inside the bags (Okoshi 2004; Okoshi and Sato-Okoshi 2011). Owing to its soft-bodied morphology, *U.major* may potentially exhibit a diminished likelihood of prosperous establishment when contrasted with organisms possessing rigid exoskeletal structures, such as select crustaceans and molluscs. Nevertheless, the surface of jute bags contains moisture and there are spaces between the shells inside, making it highly plausible that small larvae and juveniles of *U.major* measuring less than 1 cm in total length could survive within the bag. The amounts of organisms introduced into Japan from the coasts of China and the Korean Peninsula, along with imported clams, have already exceeded 10,000 tonnes; however, it is not known whether they have survived or died, except for moon snails, whose presence was manifested by eating clams. In the clam-producing areas, Matsukawa-ura transplanted clams from abroad until 2010, a year before the Great East Japan Earthquake, when they were cultivated, enlarged and shipped. The damage to clams caused by moon snails was also serious and the number of egg masses that the fishery cooperative had to exterminate each year amounted to several hundred kilograms. Considering the prolonged release of foreign clams, it is irrefutable that *U.major* may have been unintentionally introduced into Japan. Additionally, fishing bait can lead to the introduction of non-native species. In Japan, the worm bait *Perinereisaibuhitensis*, which is imported from China and other countries, has successfully established itself in certain areas (Iwasaki 2006). *U.major* is also used as fishing bait in western Japan and some areas import live organisms from China and Korea. An interview was conducted with seven fishing tackle establishments in the vicinity of Matsukawa-ura to enquire whether they had any prior experience in retailing *U.major*. Note that this interview was carried out after detailed information on the morphological characteristics had been communicated. The findings revealed that one establishment had, indeed, vended specimens of indeterminate provenance in the past. If these individuals hailed from the continental origin, there exists a potential for gene dissemination through fishing bait. Upon being released into the marine environment, these entities are commonly presumed to fall prey to a myriad of organisms, such as fish (Kaifu et al. 2013a, b). Nevertheless, there is also the likelihood of certain individuals evading predation. Furthermore, in the event that the bait individuals were bearing eggs, there is a prospect for the hatching of their larvae and subsequent dispersion of continental origin individuals within Matsukawa-ura. It is also possible that Matsukawa-ura receives *U.major* larvae from both the Kuroshio and Oyashio currents, but despite Mangoku-ura being only approximately 100 km away, continental species have not been identified there. Therefore, there is the possibility of anthropogenic introduction to Matsukawa-ura.

Conversely, in Lake Notoro, a specimen of continental origin was also detected, but there was no record of the introduction of *R.philippinarum* from China or Korea and none of the ten surrounding fishing tackle stores handled *U.major* as bait. Based on the current findings, it is highly probable that the introduction to Lake Notoro was natural and occurred via ocean currents.

### ﻿Possibility of subspecies

In this study, we focused on various morphological traits, including spines and hairs and counted, measured and compared them amongst individuals. We identified several traits that were characteristic of the comparisons amongst individuals. The divergence exhibited by MT-03, which is affiliated with Group 4, is particularly noteworthy regarding the morphology of its telson tip and section. Additionally, genetic analysis revealed a significant genetic distance of up to 0.056 from the other Japanese specimens of Groups 1, 2 and 3, indicating that it might be considered a subspecies. However, there is a lack of available morphological data for SK-02, 03, and 05, which also belong to Group 4. Therefore, further investigation is required to confirm their classification as subspecies through continuous surveys in Matsukawa-ura and Korea.

Group 4 has been proposed as the most primordial lineage that diverged within the species *U.major*, with the potential occurrence of subspecies within this particular group.

## ﻿Conclusions

We conducted morphological evaluations in conjunction with molecular phylogenetic analyses of COI genes, extracted from specimens collected in Japan, Korea, and China, to ascertain the phylogeographic patterns and genetic as well as morphological, diversity in *Upogebiamajor*. As a consequence of our analysis, *U.major* was classified into four primary groups: one with predominantly Japanese descent, two other groups inferred to have originated from the continent, and the other group genetically segregated from both Continental and Japanese descent. The group exclusively comprising Japanese specimens suggests that the planktonic larvae of this species are widely dispersed by ocean currents surrounding the Japanese Archipelago. In contrast, several Japanese specimens were included in the continental group, which may be due to the introduction of individuals from the continent via ocean currents, the possibility that this species being a continental relict or the unintentional introduction of biota imported from Korea and China. Matsukawa-ura demonstrated high genetic diversity, with specimens from all groups present. Moreover, one specimen sampled from Matsukawa-ura (MT-03) presented noteworthy genetic and morphological variances compared with the other specimens, indicating the possibility of its being a subspecies. A specimen from Gyeonggi-do, Korea, obtained from GenBank, was classified into the same group as MT-03, although information on its morphology was unavailable because the reference paper remains unpublished. To confirm these findings, further morphological and genetic investigations and analyses encompassing Matsukawa-ura and other sites are necessary.

The genetic dispersal of *U.major* suggests the existence of both natural and anthropogenic dissemination pathways, implying their intricate interplay in the shaping of regional populations. The outcomes of this study underscore the potential for analogous occurrences in all organisms, irrespective of intentional or unintentional introduction and release from neighboring regions, transcending the boundaries of this particular species. These insights not only contribute to a deeper understanding of the origins of distribution of *U.major* but also introduce a novel challenge of assessing the coexistence of these two dispersion routes.

## ﻿Additional information

### Author ORCIDs

Kyosuke Kitabatake https://orcid.org/0009-0006-4150-9622

Kentaro Izumi https://orcid.org/0000-0001-9802-071X

Natsuko I. Kondo https://orcid.org/0000-0002-0883-0506

Kenji Okoshi https://orcid.org/0000-0003-0421-2812

### Data availability

All of the data that support the findings of this study are available in the main text or Supplementary Information.

## Supplementary materials

Supplementary material 1 Molecular and morphometric analyses of *Upogebiamajor* Data type xlsx Explanation note The appendix contains certain outcomes of molecular analyses, such as variations in nucleotide numbers and genetic disparities, as well as the results and tests of morphometric analyses stratified by gender. https://binary.pensoft.net/file/923719This dataset is made available under the Open Database License (http://opendatacommons.org/licenses/odbl/1.0/). The Open Database License (ODbL) is a license agreement intended to allow users to freely share, modify, and use this Dataset while maintaining this same freedom for others, provided that the original source and author(s) are credited.Kyosuke Kitabatake, Kentaro Izumi, Natsuko I. Kondo, Kenji Okoshi
